# Patient Knowledge of GLP‐1 Receptor Agonists for Obesity and Exploration of Potential Household‐Level Effects in an Urban Health System

**DOI:** 10.1002/osp4.70199

**Published:** 2026-10-07

**Authors:** Leah M. Schumacher, Elise Rodrigues, Erin Mraz, Doria Wohler, Astha Rajpal, Sharon J. Herring, Daniel J. Rubin, Ajay D. Rao, Laura Igarabuza, Samantha Flanagan, Daniel Edmundowicz, Resa M. Jones

**Affiliations:** ^1^ Department of Social and Behavioral Sciences College of Public Health Temple University Philadelphia Pennsylvania USA; ^2^ Center for Obesity Research and Education College of Public Health Temple University Philadelphia Pennsylvania USA; ^3^ Lewis Katz School of Medicine Temple University Philadelphia Pennsylvania USA; ^4^ Section of General Internal Medicine Department of Medicine Lewis Katz School of Medicine Temple University Philadelphia Pennsylvania USA; ^5^ Department of Population Health Sciences Center for Health Justice and Bioethics Lewis Katz School of Medicine Temple University Philadelphia Pennsylvania USA; ^6^ Section of Endocrinology, Diabetes and Metabolism Department of Medicine Lewis Katz School of Medicine Temple University Philadelphia Pennsylvania USA; ^7^ Center for Biostatistics and Epidemiology Lewis Katz School of Medicine Temple University Philadelphia Pennsylvania USA; ^8^ Department of Medicine Lewis Katz School of Medicine Temple University Philadelphia Pennsylvania USA; ^9^ Family and Community Medicine Lewis Katz School of Medicine Temple University Philadelphia Pennsylvania USA; ^10^ Temple Heart & Vascular Institute Lewis Katz School of Medicine Temple University Philadelphia Pennsylvania USA; ^11^ Department of Epidemiology and Biostatistics College of Public Health Temple University Philadelphia Pennsylvania USA; ^12^ Fox Chase Cancer Center Temple Health Temple University Philadelphia Pennsylvania USA

**Keywords:** decision‐making, GLP‐1 receptor agonists, obesity, overweight, patient education, ripple effect

## Abstract

**Background:**

The use of glucagon‐like peptide‐1 receptor agonists (GLP‐1 RAs) for obesity management has gained widespread popularity and media attention. However, little is known about patients' knowledge related to GLP‐1 RAs (e.g., prescribing indications, side effects), how patients currently learn and wish to learn about GLP‐1 RAs, and how GLP‐1 RA use may impact weight‐related behaviors and attitudes within households (e.g., food purchasing patterns).

**Objective:**

This study evaluated these questions among diverse adults recruited from a large, urban health system.

**Methods:**

Participants were recruited to complete a brief online questionnaire through flyers posted in several outpatient clinics within a large health system. Recruitment occurred from November 2024 to April 2025. Descriptive statistics were calculated.

**Results:**

Fifty‐two patients completed the questionnaire. Overall, 98% of participants had heard at least “a little” about GLP‐1 RAs. The conditions participants most commonly believed could benefit from use were obesity (88%), diabetes (75%), and high blood pressure (43%). The side effects they had heard most about were nausea/vomiting (71%), diarrhea (49%), and constipation (47%). Expected amount of weight loss and understanding of GLP‐1 RAs' mechanisms of action varied widely. The most common GLP‐1 RA information source was commercials/advertisements (73%); however, the most frequently preferred information source was healthcare professionals (87%). In exploratory analyses among patients with current or past GLP‐1 RA use and ≥ 1 other household member (*n* = 12), 75% believed their use impacted adult household members' attention to weight, eating, or exercise; 50% believed GLP‐1 RA use impacted child/adolescents household members' attention to these matters; and 42% reported perceived weight loss among household adult(s). All GLP‐1 RA users reported changes in their household meal behaviors, and many reported shifts toward more healthful food purchases.

**Conclusion:**

These findings suggest that many patients understand the primary indications for GLP‐1 RAs for obesity and common side effects, while also highlighting opportunities for improved decision support. Additional research on a potential “ripple effect” within households using larger samples and more robust methods is warranted.

## 1 Introduction

Glucagon‐like peptide‐1 receptor agonists (GLP‐1 RAs) and glucose‐dependent insulinotropic polypeptide (GIP)/GLP‐1 dual receptor agonists—collectively referred to herein as GLP‐1 RAs—have shown great success in promoting weight loss among those with obesity [1, 2]. These medications can also improve glycemic control in patients with type 2 diabetes (T2D) and support cardiovascular health, among other benefits [1, 2]. The American Diabetes Association recommends GLP‐1 RAs as first‐line pharmacotherapy in patients with T2D and overweight or obesity [3]. Similarly, the American Heart Association highlights GLP‐1 RAs, including semaglutide, as effective treatments for obesity that can improve cardiovascular health [4]. As of 2023, Medicaid coverage of GLP‐1 RAs has expanded to 1 in 5 states [5]. Currently, the Medicare GLP‐1 Bridge program provides eligible Medicare Part D recipients access to certain GLP‐1 RAs for weight loss/obesity management for a $50 monthly copay [6].

As research continues to establish the broad benefits of GLP‐1 RAs, discourse on them has proliferated. A poll published in July 2026 found that 11% of American adults reported currently using a GLP‐1 RA for weight loss, with 15% having used one for this purpose at some point [7]. Worldwide, online searches for “semaglutide” have increased exponentially since 2022, coinciding more with media reporting and social media attention on the topic than with country‐specific drug administration approval of the medication [8]. Patient motivations for taking GLP‐1 RAs, barriers to their use (e.g., supply shortages, costs, stigma), and patient experiences with these medications have also received attention in the media and scientific literature [9, 10, 11, 12, 13, 14, 15, 16, 17].

While these findings offer important information about public perceptions of and patient experiences with GLP‐1 RAs, [9, 10, 11, 12, 13, 14, 15, 16, 17] fewer studies have focused on patients' knowledge about GLP‐1 RA indications and their potential risks and benefits—important considerations for patients who want to use these medications or may be good candidates for doing so. Little is also known about how patients prefer to learn about these medications. Research on these topics has been especially scarce among populations at highest risk for obesity and related conditions like T2D and cardiovascular disease, such as individuals identifying as Non‐Hispanic Black or Hispanic and those with lower educational attainment [18, 19]. Understanding the extent and accuracy of these patient populations' knowledge of GLP1‐RAs and how they wish to learn more is important to avoid perpetuating disparities in clinical care and outcomes [20]. Additionally, while a “ripple effect” has been described for metabolic and bariatric surgery and lifestyle modification programs, [21, 22] the impact of GLP‐1 RA use on weight‐related conversations, attitudes, and behaviors within households is only beginning to be studied [15, 16, 17, 23]. Results to date suggest that many parents (i.e., 50% in one study [17]) who take GLP‐1 RAs talk with their children about their decision to use this medication, [15, 17] and that parental GLP‐1 RA use may have several effects on children's weight‐related behaviors and attitudes [15, 16, 17]. However, little is known about how GLP‐1 RA use may affect adult household members, and there are only a handful of studies on this emerging topic, warranting further investigation.

This study explored patients' awareness of and the accuracy of their knowledge of GLP‐1 RAs (Aim 1), how patients prefer to learn about GLP‐1 RAs (Aim 2), and the potential impact of a patient's GLP‐1 RA use on several aspects of weight‐related attitudes and behaviors within the home (Aim 3). Prior GLP‐1 RA use was also assessed and was considered when examining response patterns across aims. The study examined these questions in several clinics at a large, urban health system with a racially and ethnically diverse patient population, a substantial portion of whom have lower income and/or lower educational attainment [24]. This population has a higher prevalence of obesity than other segments of the general population [18, 19] and has demographics that differ from those in many previous surveys on GLP‐1 RAs (e.g., a greater proportion of patients identifying as Non‐Hispanic Black or Hispanic) [13, 14, 25]. By focusing on this specific population, we sought to elucidate the needs of a patient population who may greatly benefit from education about GLP‐1 RAs and improved access to GLP‐1 RAs, work that is highly relevant for clinicians and researchers who work in safety‐net clinics and health systems.

## 2 Methods

### 2.1 Study Design and Setting

This was a cross‐sectional study. Patients were recruited through flyers placed in the clinic waiting rooms (e.g., at the check‐in desk, on bulletin boards, on tables with other literature) and/or in the exam rooms (e.g., posted on the wall) in four outpatient clinics (Family and Community Medicine; General Internal Medicine; Endocrinology, Diabetes and Metabolism; Heart and Vascular Institute) at Temple Health's main campus in North Philadelphia, which serves a large proportion of historically marginalized, medically vulnerable (or medically underserved) patients and their families. Front desk staff and collaborating providers at the clinics were also encouraged to direct patients' attention to the flyers, although this was not required to avoid concerns around potential coercion and to respect differences in clinical workflows and demands. Flyers included information about the questionnaire, along with a QR code and URL link that directed prospective participants to a brief eligibility screener and the online informed consent form. Each clinic's flyers had a unique QR code and URL link, allowing for tracking of responses by clinic. After providing informed consent online, participants received an emailed link to complete an online REDCap questionnaire. Self‐reported data were collected from November 2024 to April 2025. Recruitment was closed after approximately 5 months to limit potential temporal trends in findings given the rapidly changing GLP‐1 RA landscape. Participants received a $15 Amazon gift card for completing the 10–15 min questionnaire. The study was approved by the Temple University Institutional Review Board.

### 2.2 Participants

Eligibility required patients to be 18 years or older per self‐report, to have access to the internet and an email account to complete the survey, and to complete the consent form and questionnaire in English. There were no exclusion criteria. Eligibility criteria were purposefully left broad to elicit a broad range of perspectives on GLP‐1 RAs.

### 2.3 Measures

A literature search of PubMed and Google Scholar was conducted to identify studies related to GLP‐1 RA use and the measures/questions previously used to assess constructs of interest. The study questionnaire consisted of questions adapted from several of these sources, including recent KFF and Pew surveys about GLP‐1 RAs [14, 26] and previous peer‐reviewed studies [27, 28, 29, 30]. Branching logic was used to ensure that participants only responded to questions for which they had appropriate knowledge or experiences (e.g., previous GLP‐1 RA use).

GLP‐1 RA Awareness: All participants responded to the question: “How much have you heard about a new class of medications being used for weight loss, such as Ozempic, Wegovy, Mounjaro, and Zepbound? These drugs are sometimes called GLP‐1 agonists.” Response options were *Not at all*, *A little*, *Some*, or *A lot* [26]. All participants who indicated hearing at least “a little” about these medications then responded to several questions assessing how much of what they had heard about GLP‐1 RAs had focused on topics such as weight loss, the treatment or prevention of health conditions, celebrities using the medications, and cost/insurance coverage (response options: *Not at all*, *A little*, *Some*, or *A lot*).

GLP‐1 RA Knowledge: All participants who had heard at least “a little” about GLP‐1 RAs reported what conditions they had heard can be treated by these medications (select all format, e.g., *Overweight or obesity*, *Diabetes*, *Sleep apnea*); expected weight loss at 3, 6, and 12 months (response options: 0–25, 25–50, 50–75, 75–100, 100+ lbs); and potential side effects (select all format, e.g., *Nausea and/or vomiting, Headache*, *Constipation*). Participants also reported how much they had heard about GLP‐1 RAs' physiological mechanisms of action (response options: *Not at all*, *A little*, *Some*, or *A lot*). Those who reported hearing at least “a little” were asked to share what they had heard using a free‐text response.

Learning Preferences: Participants who had heard at least “a little” about GLP‐1 RAs were asked to report sources from which they had received information on GLP‐1 RAs (select all format, e.g., *Commercials or advertisements*, *Family members*, *Doctor or other healthcare professional*s). All participants were asked to report their interest in learning more about GLP‐1 RAs (response options: *Not at all*, *A little*, *Some*, or *A lot*) and their preferred source(s) for learning about health information (select all format, e.g., *Commercials or advertisements*, *Family members*, *Doctor or other healthcare professional*s).

Patient Health and Prior GLP‐1 RA Use: Participants self‐reported the presence of several obesity‐related conditions (select all format, e.g., *Diabetes*, *Osteoarthritis*, *High blood pressure*) and their perceived weight status (response options: *Very underweight*, *Slightly underweight*, *About the right weight*, *Slightly overweight*, *Very overweight*) [14]. Participants also reported whether they had ever used a GLP‐1 RA (response options: *Yes, currently using*; *Yes, used in the past but not currently using*; *No, never*) and if so, which one (response options: *Ozempic*, *Wegovy*, *Mounjaro*, *Zepbound*, “*other*” *[specify]*).

Household Effects: Participants reported on the number of adults and/or children/adolescents (< 18 years) residing in their household over the past 3 months. Participants with current or past GLP‐1 RA use and at least one other adult household member then reported how much they believed taking a GLP‐1 RA had influenced adult household members to think about their (1) weight, (2) eating habits, and (3) exercise habits (response option for each: *Not at all, A little, Some, A lot*). Participants with children/adolescents household members repeated the same set of questions for these individuals collectively. Participants were also asked whether they believed any adult household members had lost weight since the participant began taking a GLP‐1 RA (response options: *Yes*, *No*, *Unsure*, *Prefer not to answer*); once again, this question was then repeated for children/adolescents, as appropriate. Lastly, participants reported any changes in purchasing of several types of foods such as “fruits and vegetables,” “Dairy products,” and “Breads, pasta, rice, cereals” (response options: *Decreased*, *Increased*, *No change*) [30]. They also reported any changes in household meal/eating patterns using a select all format (example items: *We eat smaller meals*; *I eat fewer meals or at different times than my household members*; *We eat more meals at home*). These items were adapted from prior research, [29] with revisions/additions made by the research team in an effort to capture the range of possible changes to meal/eating patterns associated with GLP‐1 RA use per anecdotal patient reports to clinical members of the study team and experiences shared in the mass media.

Demographics: Participants self‐reported age, gender, racial and/or ethnic identity (select all format, e.g., *Black or African American*, *White*, *Hispanic or Latino)*, household income (using prespecified ranges, e.g., *Less than $20,000*; *$30,000 to less than $40,000*; *$100,000 to less than $200,000*), type of health insurance (example response options: *Plan through my employer or my spouse's employer; Medicaid, the government program for certain low‐income adults and children*), and perceived ease of paying for prescription medications in general (response options: *Very easy*, *Somewhat easy*, *Neither easy nor difficult*, *Somewhat difficult*, *Very difficult*, *I do not have any prescriptions medications*) [25, 26].

### 2.4 Data Analysis

All data were screened for participant uniqueness, item completion, and implausible response patterns. To ensure that these data were reported from humans and not bots, and to prevent duplicate responses from the same participant given the fully online format, we put several safeguards in place. If any participant completed more than one questionnaire, only the first questionnaire was used. Further, only complete surveys were considered valid and any completion time < 2 min was considered implausible given the required time to complete the questionnaire based on pre‐testing. See Supplemental Materials for details on participant flow.

All three aims were evaluated using descriptive statistics. Most variables were characterized using counts and percentages. Age was characterized with mean and standard deviation. Given the smaller sample size, which is commensurate with other recent research on patients' experiences with GLP‐1 RAs, [15] between‐group differences for participants with versus without prior GLP‐1 RA use were not compared statistically. However, for some aims, data are presented separately by groups for hypothesis‐generating purposes.

## 3 Results

### 3.1 Demographics

The survey was completed by 52 individuals. As shown in Table 1, most participants identified as women (85%). The mean age was 38.9 years (standard deviation = 11.9 years). Forty percent of participants were Hispanic, 27% were Black, and 21% were White. Nearly half (44%) had a household income under $50,000 annually. The most common health insurance types were employer‐sponsored (56%) and Medicaid (19%). Approximately 80% of participants reported being slightly or very overweight, and 71% reported that a health care provider had told them they had overweight or obesity. The other top chronic conditions endorsed were high blood pressure (39%), high cholesterol (25%), and diabetes (23%). For the recruitment clinic, most respondents were from Endocrinology, Diabetes and Metabolism (63%), followed by the Heart and Vascular Institute (15%), Family and Community Medicine (14%), and General Internal Medicine (8%). Twenty‐one respondents (40%) reported current or prior GLP‐1 RA use, while 31 (60%) reported no history of use.

**TABLE 1:** Participant sociodemographic characteristics (*N* = 52).

		Count (%)
Gender	Woman	44 (85%)
	Man	6 (12%)
	Non‐binary	0 (0%)
	Other	0 (0%)
Race and/or ethnic identity (select all format)^a^	Black or African American	14 (27%)
	Hispanic or Latino	21 (40%)
	Native Hawaiian or Other Pacific Islander	0 (0%)
	White	11 (21%)
	American Indian/Alaska Native; Asian; or Middle Eastern or North African^a^	5 (10%)
	Prefer to self‐describe	0 (0%)
	Prefer not to answer	4 (8%)
Total household income before taxes	Less than $20,000	9 (17%)
	$20,000 to less than $50,000	14 (27%)
	$50,000 to less than $75,000	11 (21%)
	$75,000 to less than $100,000	8 (15%)
	$100,000 to less than $200,000	4 (8%)
	$200,000 or more	3 (6%)
How easy or difficult is it to pay for prescriptions	Very easy	12 (23%)
	Somewhat easy	11 (21%)
	Neither easy nor difficult	6 (12%)
	Somewhat difficult	9 (17%)
	Very difficult	8 (15%)
	I do not have any prescription medications	0 (0%)
Type of health insurance	Private employer‐sponsored plan	29 (56%)
	Medicare	4 (8%)
	Medicaid	10 (19%)
	Self‐purchased	4 (8%)
	Uninsured	2 (4%)
	Other (e.g., plan through parents)	3 (6%)
Self‐perceived weight status	Very underweight	2 (4%)
	Slightly underweight	1 (2%)
	About the right weight	7 (14%)
	Slightly overweight	24 (46%)
	Very overweight	17 (33%)
Presence of chronic conditions (select all)	Overweight/obesity	37 (71%)
	Diabetes	12 (23%)
	Heart disease	2 (4%)
	High blood pressure	20 (39%)
	High cholesterol	13 (25%)
	Stroke	0 (0)%
	Kidney disease	1 (2%)
	Sleep apnea	11 (21%)
	Metabolic dysfunction‐associated steatotic liver disease	3 (6%)
	Osteoarthritis	3 (6%)
	None of these	7 (14%)

^a^ Cells for the response options “American Indian/Alaska Native,” “Asian,” or “Middle Eastern or North African” were combined due to low rates of endorsement to avoid potential identification.

### 3.2 Awareness

Overall, 98% (*n* = 51) of participants reported having heard at least “a little” about GLP‐1 RAs. As shown in Table 2, for the overall sample, the top three topics that participants had heard “a lot” or “some” about were weight loss (94% of participants), treatment or prevention of health conditions (76%), and difficulty finding GLP‐1 RAs at pharmacies due to shortages (71%). Participants had heard the least about potential long‐term health problems these medications may cause (35% of participants) and the potential of GLP‐1 RAs to lower cravings for alcohol (43%) and cigarettes (29%). Table 2 also displays response patterns for those with (*n* = 21) versus without (*n* = 30) prior GLP‐1 RA use (note: one individual without prior GLP‐1 RA use reported never hearing of GLP‐1 RAs, so they were not asked to respond to these questions).

**TABLE 2:** Topics participants had heard about pertaining to GLP‐1 RAs: Full sample and stratified by GLP‐1 RA use.

	Context (participants have heard some or a lot about the following.)	Full sample (*N* = 51)^a^		Never user of GLP‐1 RAs (*n* = 30)		Current or former user of GLP‐1 RAs (*n* = 21)	
		*n*	%	*n*	%	*n*	%
How much of what you have heard about these medications has focused on the following?	Weight loss	48	94	28	93	20	95
	Treatment or prevention of health conditions	39	76	20	67	19	90
	Celebrities using these medications to become thin	33	65	19	63	14	67
	Side effects	27	53	13	43	14	67
	Long‐term health problems they might cause	18	35	6	20	12	57
	Cost	34	67	17	57	17	81
	Health insurance coverage	35	69	18	60	17	81
	It being hard to find these medications at a pharmacy because of shortages	36	71	20	67	16	76
	Potential to reduce food noise (frequent thoughts about food and eating)	32	63	16	53	16	76
	Potential to lower cravings for alcohol	22	43	9	3	13	62
	Potential to lower cravings for cigarettes	15	29	5	17	10	48
	Other (specify)	4	8	1	3	3	14

^a^ 1 participant had not heard about GLP‐1 RAs and did not answer this question.

### 3.3 Knowledge

Indications: Table 3 shows the condition participants believed could benefit from GLP‐1 RA use, both for the full sample and those with versus without prior use. For the full sample, the conditions chosen most often were overweight or obesity (88%), diabetes (75%), and high blood pressure (43%), while the least chosen conditions were osteoarthritis (2%), metabolic dysfunction‐associated steatotic liver disease (previously known as nonalcoholic fatty liver disease, NAFLD) (8%), and kidney disease (20%). Response patterns appeared similar for participants who had never taken a GLP‐1 RA and those with current or previous GLP‐1 RA use (e.g., the same 5–6 conditions were endorsed most frequently in both groups, few individuals in both groups endorsed conditions like osteoarthritis). However, exact percentages of endorsement and rank varied.

**TABLE 3:** Health conditions participants believed could benefit from GLP‐1 RA use: Full sample and stratified by GLP‐1 RA use.

Based on what you have heard, what are health conditions that can be prevented or treated by this new class of medications including Ozempic, Wegovy, Mounjaro, and Zepbound?	Full sample (*N* = 51)^a^		Never users (*n* = 30)		Current and former users (*n* = 21)	
	*n*	%	*n*	%	*n*	%
Overweight or obesity	45	88	25	83	20	95
Diabetes	38	75	18	60	20	95
Heart disease (ex: heart attack, heart failure)	20	39	11	37	9	43
High blood pressure	22	43	14	47	8	38
High cholesterol	18	35	12	40	6	29
Stroke	12	24	9	30	3	14
Kidney disease	10	20	6	20	4	19
Sleep apnea	13	25	7	23	6	29
Metabolic dysfunction‐associated steatotic liver disease	4	8	3	10	1	5
Osteoarthritis	1	2	1	3	0	0
Other	1	2	0	0	1	5
Unsure	4	8	3	10	1	5

*Note: n* depicts the number of participants who selected this response.

^a^ 1 participant had not heard about GLP‐1 RAs and did not answer this question.

Weight Loss Expectations: Participants' expected weight losses over time are shown in Table 4. At 3 months, most participants (69%) believed that a person could lose 0–25 lbs. At 6 months, nearly half (48%s) believed a person could lose 25–50 lbs. At 12 months, 30% of participants believed a person could lose 25–50 lbs. and 34% believed a person could lose 50–75 lbs. While most participants who had never taken a GLP‐1 RA and most participants with current or previous GLP‐1 RA use believed that a person could lose approximately 0 and 25 lbs at 3 months (73% of never users and 62% of current/former users), responses for how much a person could lose at 6 and 12 months varied (Table 4).

**TABLE 4:** Expected weight loss from GLP‐1 RA use over time: Full sample and stratified by GLP‐1 RA use.

How much weight do you think a person typically loses when taking this new class of medications including Ozempic, Wegovy, Mounjaro, and Zepbound?				
		Weight loss at 3 months, *n* (%)	Weight loss at 6 months, *n* (%)	Weight loss at 12 months, *n* (%)
Full sample (*N* = 51)^a^	0–25 lbs	35 (69)	13 (26)	5 (10)
	25–50 lbs	6 (12)	24 (48)	15 (30)
	50–75 lbs	5 (10)	6 (12)	17 (34)
	75–100 lbs	0 (0)	3 (6)	8 (16)
	More than 100 lbs	1 (2)	0 (0)	1 (2)
	Unsure	4 (8)	4 (8)	4 (8)
Current & formers users (*n* = 21)^b^	0–25 lbs	13 (62)	7 (35)	1 (5)
	25–50 lbs	5 (24)	7 (35)	9 (45)
	50–75 lbs	2 (10)	4 (20)	3 (15)
	75–100 lbs	0 (0)	2 (10)	7 (35)
	More than 100 lbs	1 (5)	0 (0)	0 (0)
	Unsure	0 (0)	0 (0)	0 (0)
Never users (*n* = 30)	0–25 lbs	22 (73)	6 (20)	4 (13)
	25–50 lbs	1 (3)	15 (57)	6 (20)
	50–75 lbs	3 (10)	2 (7)	14 (47)
	75–100 lbs	0 (0)	1 (3)	1 (3)
	More than 100 lbs	0 (0)	0 (0)	1 (3)
	Unsure	4 (13)	4 (13)	4 (13)

*Note:* One additional participant had missing data for 6 and 12 months.

^a^ 1 participant had not heard of GLP‐1 RAs and did not answer this question.

^b^ 1 participant was missing data for 6 and 12 months.

Side Effects: Table 5 presents data on side effects for the full group and among those with and without prior GLP‐1 RA use. In the full sample, the most common side effects that participants had heard about were nausea and/or vomiting (71% of participants), diarrhea (49%), and constipation (47%). The side effects that participants had heard about least were unplanned pregnancy (10% of participants), drowsiness (12%), and depression and/or mood swings (14%). Response patterns appeared quite similar across participants who had never taken a GLP‐1 RA and those with current or previous GLP‐1 RA use (e.g., the same 4 side effects were endorsed most frequently in both groups). However, for most items, rates of endorsement appeared higher among those with current or former GLP‐1 RA use.

**TABLE 5:** Side effects participants believed could occur with GLP‐1 RA use: Full sample and stratified by GLP‐1 RA use.

Based on what you have heard, what are side effects of these medications?	Full sample *N* = 51^a^		Never users (*n* = 30)		Current and former users (*n* = 21)	
	*n*	%	*n*	%	*n*	%
Nausea and/or vomiting	36	71	17	57	19	91
Diarrhea	25	49	11	37	14	67
Constipation	24	47	10	33	14	67
Depression and/or mood swings	7	14	4	13	3	14
Drowsiness	6	12	1	3	5	24
Headache	16	31	9	30	7	33
Unplanned pregnancy	4	10	1	3	3	14
Other (specify)	3	6	3	10	0	0
Unsure	3	6	3	10	0	0

*Note: n* depicts the number of participants who endorsed this response.

^a^ 1 participant had not heard about GLP‐1 RAs and did not answer this question.

Mechanism of Action: Overall, 26% (*n* = 13) of participants had heard “nothing” about GLP‐1 RAs' mechanisms of action; conversely, 26% had heard “a lot” about mechanisms of action (26%, *n* = 13). Twenty‐three participants provided a free‐text response describing in their own words how GLP‐1 RAs work in the human body. Of these, 44% (*n* = 10) described GLP‐1 RAs as causing reduced hunger, 17% (*n* = 4) described GLP‐1 RAs as causing weight loss, and 17% (*n* = 4) described GLP‐1 RAs as slowing down a person's metabolism or replicating natural GLP‐1 receptors in the brain.

### 3.4 Learning Preferences

The top sources of current information about GLP‐1 RAs were commercials or advertisements (73%, *n* = 37), doctor or other healthcare professionals (63%, *n* = 32), and mass media or social media (53%, *n* = 27). Participants varied in their level of interest in learning more about GLP‐1 RAs; 29% (*n* = 15) were “not at all,” 19% (*n* = 10) were “a little,” 29% (*n* = 15) were “moderately,” and 23% (*n* = 12) were “extremely” interested in learning more. Most participants reported preferring to learn about their health from a physician or other healthcare professional (87%, *n* = 45), followed by academic resources such as health articles online (31%, *n* = 16), commercials or advertisements (21%, *n* = 11), family members (21%, *n* = 11), mass media or social media (19%, *n* = 10), and friends or community members (13%, *n* = 7).

### 3.5 Household‐Level Effects

Figure 1 shows the number of participants with other household members, stratified by GLP‐1 RA use status and whether participants had adults and/or children/adolescent household members.

**FIGURE 1:**
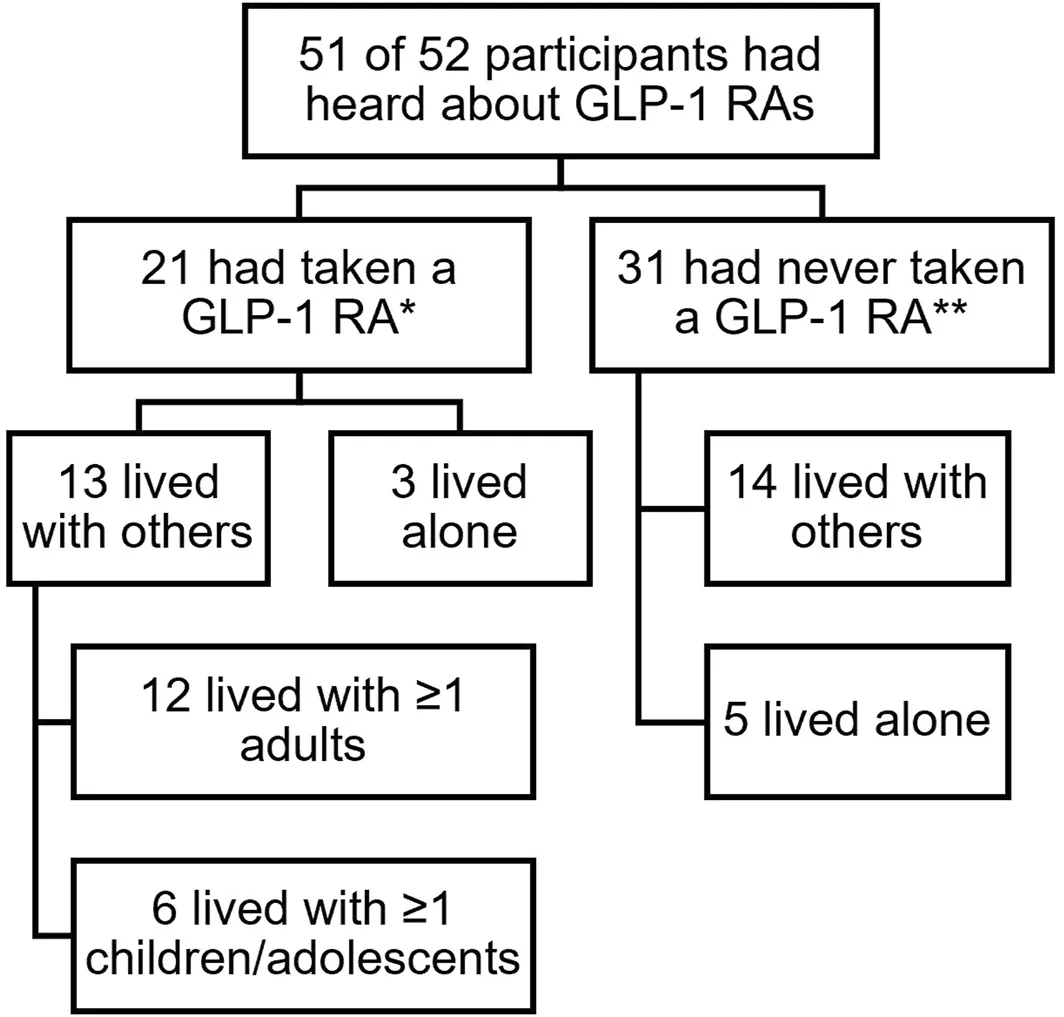
Breakdown of respondents by GLP‐1 RA use and household composition. *Of the 21 participants who had taken a GLP‐1 RA, 5 left the question about the number of household members blank. Additionally, of those who reported having household members, 1 left the question about the ages of their household members blank. **Of the 31 participants who had never taken a GLP‐1 RA, 10 left the question about the number of household members blank. Additionally, of those who reported having household members, 1 left the question about the ages of their household members blank.

Among the 12 participants with current or past GLP‐1 RA use and at least one reported adult household member, 75% (*n* = 8) reported that taking a GLP‐1 RA had influenced adult members of their household to think about their own weight, eating habits, and physical activity either “some” or “a lot.” For the 6 participants with current or past GLP‐1 RA use and at least one reported child/adolescent household member, 50% (*n* = 3) reported that taking a GLP‐1 RA had influenced the children or adolescents in their household to think about their own weight, eating habits, and physical activity “some.” No participant reported that taking a GLP‐1 RA had “a lot” of influence on these outcomes for children/adolescents.

For household member weight loss, 42% (*n* = 5) of the 12 participants reported that at least one adult household member had lost weight since the respondent started a GLP‐1 RA; 33% (*n* = 4) of participants said no, 8% (*n* = 1) was unsure, and 17% (*n* = 2) declined to answer. No participant reported that child/adolescent household members had lost weight; four participants said no, one was unsure, and one declined to answer.

For changes in household food purchasing patterns since GLP‐1 RA initiation, the food categories that decreased most often were “baked goods, crackers, chips, snacks” (81% of participants reported a decrease, *n* = 17); “breads, pasta, rice, cereals” (76% reported a decrease, *n* = 16); and “beverages (e.g., soda, fruit juice)” (62% reported a decrease, *n* = 13). The food categories that increased most often were “fruits and vegetables” (71% of participants reported an increase, *n* = 15) and “poultry and seafood” (48% reported an increase, *n* = 10). The food categories where participants most often reported no change in purchasing habits were “miscellaneous food items (e.g., condiments)” (endorsed by 57%, *n* = 12), “dairy products” (48%, *n* = 10), and “red meat” (38%, *n* = 8).

Regarding changes in household meal behaviors, 69% (*n* = 9) of participants reported that their household ate healthier food, 46% (*n* = 6) reported that their household ate smaller meals, and 46% (*n* = 6) reported that they ate different types of foods than their household members at meals. All participants endorsed at least one change in their household meal behaviors.

## 4 Discussion

This study was among the first to examine patients' knowledge of GLP‐1 RAs, their preferences for learning more about GLP‐1 RAs, and the potential effect of GLP‐1 RAs on weight‐related behaviors and attitudes within the home. This study was also conducted among a diverse patient population at a large, urban health system in a historically underserved and under‐resourced area of Philadelphia. Given that obesity disproportionately impacts individuals of historically minoritized racial and ethnic identities [19]—as well as the high prevalence of obesity and related conditions in this sample—the current findings may offer insights into potential informational needs and preferences for a patient population that has been less studied for GLP‐1 RA perceptions but could benefit greatly from their use.

Aligning with national trends, nearly all study participants had heard at least a little about GLP‐1 RAs [7, 25]. Building on previous work, we also found that most participants' understanding of the indications for GLP‐1 RAs aligned with FDA‐approved indications. Similarly, the side effects most participants had heard about are among the most common in clinical trials [2, 31]. These findings may have been influenced by the fact that a large majority of respondents were coming from specialty clinics (e.g., endocrinology), where they may have had more exposure to GLP‐1 RAs given these medications' history of use for diabetes control. These findings should also be interpreted with the study's convenience sampling approach and modest response rate in mind, as patients who knew more about GLP‐1 RAs may have been more likely to respond (see discussion of limitations below). Still, they are encouraging as they suggest that patients maintained reasonably accurate knowledge of these topics regardless of where they obtained information. We observed lower patient knowledge of lesser‐known indications and potential side effects. For these indications and concerns, education may be most effective when it is patient‐specific and when appropriate counseling can be provided. For example, some patients may benefit from certain GLP‐1 RAs as part of their kidney disease treatment or for the management of existing cardiovascular disease [32, 33]. Patients with a history of mental illness may benefit from knowing that some individuals have reported worsening mental health symptoms on GLP‐1 RAs, although data are mixed and the FDA recently requested the removal of a suicide ideation and behavior warning from GLP‐1 RAs' label [34, 35, 36, 37]. Notably, participants had heard a lot about how difficult it can be to find GLP‐1 RAs at pharmacies. This highlights potential accessibility issues of these medications, as well as the role providers can play in keeping patients updated on changes in medication access, cost, and coverage—all of which may shift frequently.

Our findings also offered insights into patients' expectations for weight loss with GLP‐1 RAs and their understanding of these medications' mechanisms of action. When patients know more about their medications, they are more likely to adhere to their medication regimen, monitor for potentially dangerous side effects, and better manage their conditions [38, 39]. When asked how much weight patients thought could be lost at various time points, patients' responses varied widely. This suggests that it may be helpful for providers to review typical weight losses for each agent to ensure appropriate expectations around weight loss. Of note, most literature reports weight loss with GLP‐1 RAs as a percentage of a patient's weights [40]. However, our survey asked participants how much they thought a person could lose in absolute pounds, as that is the unit individuals may be most accustomed to thinking in. The need to convert trial‐based weight loss metrics into units more intuitive for patients may underlay some of the atypically large expected weight reductions reported (e.g., > 100 lbs). Exposure to more extreme cases on social media and elsewhere may also have influenced expectations [41]. Additionally, over a quarter of participants in our survey had heard little or nothing about how GLP‐1 RAs work, highlighting the need for better patient education efforts.

While most participants in our survey preferred to learn about medications from their doctor, most had learned about these medications through a combination of advertisements, social media, and healthcare professionals. Among participants who had taken GLP‐1 RAs, 90% had heard about these medications from their doctor, versus 42% among never users. Our survey highlights the potential for improved education efforts by healthcare professionals who prescribe GLP‐1 RAs, as ideally all patients who have taken GLP‐1 RAs would be informed about them by their doctor. Additionally, many patients who could benefit from GLP‐1 RAs may not do not hear about them from providers, potentially hindering patients' consideration of this approach. Additional GLP‐1 RA education efforts by healthcare professionals may be especially important if the individuals who responded to our survey were those with greatest knowledge of or interest in GLP‐1 RAs—a possibility we cannot rule out given our convenience sampling approach.

While our findings about potential household‐level effects should be viewed as very preliminary and hypothesis‐generating given our small sample size, our study suggests that adult household members of a GLP‐1 RA user may be more likely to think about their own weight, eating habits, and exercise habits—and even lose weight. In contrast, participants perceived a more limited impact of their GLP‐1 RA use on child or adolescent household members for these outcomes. These exploratory findings build on several recent studies that have looked at the potential impact of GLP‐1 RA use on weight‐related conversations, attitudes, and practices of others in the home but have focused primarily on the implications of parental GLP‐1 RA use on children and adolescents [15, 16, 17]. Participants also reported an impact of their GLP‐1 RA use on grocery shopping, such that the purchasing of several less nutritious types of food decreased (e.g., sugar‐sweetened beverages), while the purchasing of more healthful foods (e.g., fruits and vegetables) tended to increase. These findings complement recent work showing a GLP‐1 RA‐related decrease in grocery spending, especially on calorie‐dense, processed foods, as well as a decrease in spending at fast food chains, coffee shops, and limited‐service restaurants [42]. Most participants who had taken GLP‐1 RAs also reported changes in meal eating patterns (e.g., the household eating healthier food or smaller meals), demonstrating that behavioral changes associated with GLP‐1 RA use may be shared among the household. Similar changes in household meal patterns have been observed after other types of weight loss treatment and have recently been observed in other samples using GLP‐1 RAs, warranting further research on this phenomenon [15, 16, 17, 22, 23, 43].

Strengths of the study included investigation of novel, clinically meaningful aspects of patients' experiences with GLP‐1 RAs; recruitment of a racially and ethnically diverse sample whose identities align with those often disproportionately impacted by obesity; and use of measures that have been previously used or that were adapted from previously used questions, facilitating comparison with the broader literature. Several limitations should also be noted. The sample was quite small, especially for certain subsets of questions (e.g., household‐related questions); convenience sampling was used; and many respondents came from specialty clinics rather than primary care. Additionally, the number of survey respondents was lower than anticipated. While further data collection would be required to clarify and confirm reasons for the modest response rate, it is possible our recruitment approach was too passive to gain patients' attention (e.g., patients were distracted by other tasks in the clinic), an inappropriate incentive structure (e.g., patients did not consider a $15 gift card adequate), technological challenges related to the survey being available only online and involving multiple (though brief) steps, or some combination thereof. It is also possible some patients were not interested in the topic, although intense public interest in GLP‐1 RAs [7] partially assuages this concern. However, our findings should be interpreted in light of these considerations since findings may not generalize to all patients. Studies with larger samples that utilize alternative sampling frames, recruitment approaches, and incentive structures are warranted.

Additionally, all data were self‐reported (e.g., weight status, household effect outcomes); some participants elected to skip certain questions, resulting in missing data; and some questions were revised from existing measures (e.g., meal pattern questions) or created de novo (e.g., weight loss expectations). Studies that utilize alternative measurement methods (e.g., objective measurement of weight change, observation of household behaviors, interviews to collect data on topics perceived as sensitive) could refine and deepen our understanding of these topics. Further assessment of how social drivers of health may impact GLP‐1 RA interest, knowledge, and use is also important, as is evaluation of moderators that may influence the impact of GLP‐1 RAs within households (e.g., the age, weight status, and existing health habits of household members; whether household members are on GLP‐1 RAs or have sought other forms of obesity treatment like metabolic and bariatric surgery; the culture around weight and health within the home) [15, 16, 17]. These factors, some of which have been shown to be important for understanding GLP‐1 RAs' household‐level impact in recent work [15, 16, 17], may be critical for informing intervention approaches that maximize health benefits while minimizing risks (e.g., unintentional contributions to unhealthy weight control practices or disordered eating).

In summary, newer GLP‐1 RAs have shown great promise for weight loss and are recommended by several professional organizations for the management of obesity and obesity‐related comorbidities. These medications have become a dominant topic in the media, and national polls have consistently shown that the majority of Americans are familiar with them. Our study demonstrated that participants understood the primary indications for GLP‐1 RA use and common side effects, but had less understanding of lesser‐known indications and possible side effects. Participants preferred to learn about these medications from their doctor; however, many instead received information from social media and advertisements. Lastly, our data add to the growing literature on a possible household impact on weight and dietary/activity behaviors associated with GLP‐1 RA use, including by highlighting a potential effect on adult household members. Future studies may focus on better understanding patients' motivations for taking GLP‐1 RAs and reasons for discontinuation, evaluate the effectiveness of certain patient education tools, and better elucidate the effect GLP‐1 RAs may have on households.

## Author Contributions

L.M.S. conceptualized the study, with assistance from E.R., S.J.H., D.J.R., A.J.R., L.I., S.F., D.E., and R.M.J., L.M.S. oversaw data collection. E.M., D.W., A.R., S.J.H., D.J.R., A.J.R., L.I., S.F., and D.E. assisted with data collection. L.M.S., E.R., and R.M.J. led data analysis. L.M.S. and E.R. wrote the initial version of the manuscript. E.R. created the tables. All authors assisted with data interpretation and manuscript editing. All authors have read and approved the final version of the manuscript.

## Funding

This work was supported by funds from Temple University's College of Public Health.

## Conflicts of Interest

L.M.S. reports support from The Obesity Society and the International Federation for the Surgery of Obesity and Metabolic Disorders for attending meetings and/or travel; editorial board membership for Surgery for Obesity and Related Diseases; and Publications Committee membership for The Obesity Society. A.D.R. reports support for attending meetings and/or travel from Monarch Medical Technologies, and participation on a Data Safety Monitoring Board or Advisory Board for Spruce Biosciences and Concept Inc. S.F. reports payment or honoraria from the American Board of Obesity Medicine–Item Writing Committee, American College of Osteopathic Family Physicians–Module Editor, France Foundation–Module Editor, and C.M.E. Presentations. E.R., E.M., D.W., A.R., S.J.H., D.J.R., L.I., D.E., and R.M.J. report no potential conflicts of interest.

## Supporting information

Figure S1: Study flow diagram.

## Data Availability

The data that support the findings of this study are available from the corresponding author upon reasonable request.

## References

[1] K. H. Sheahan , E. A. Wahlberg , and M. P. Gilbert , “An Overview of GLP‐1 Agonists and Recent Cardiovascular Outcomes Trials,” Postgraduate Medical Journal 96, no. 1133 (2020): 156–161, 10.1136/postgradmedj-2019-137186. PMC7042958 31801807

[2] L. Liu , Z. Li , W. Ye , et al., “Safety and Effects of Anti‐obesity Medications on Weight Loss, Cardiometabolic, and Psychological Outcomes in People Living With Overweight or Obesity: A Systematic Review and Meta‐Analysis,” EClinicalMedicine 79 (2025): 103020, 10.1016/j.eclinm.2024.103020. PMC11743856 39834714

[3] American Diabetes Association Professional Practice Committee , “8. Obesity and Weight Management for the Prevention and Treatment of Diabetes: Standards of Care in Diabetes–2026,” supplement, Diabetes Care 49, no. S1 (2026): S166–S182, 10.2337/dc26-S008. PMC12690172 41358882

[4] D. Laddu , I. J. Neeland , M. Carnethon , et al., “Implementation of Obesity Science Into Clinical Practice: A Scientific Statement From the American Heart Association,” Circulation 150, no. 1 (2024): e7–e19, 10.1161/cir.0000000000001221. PMC11416804 38766861

[5] B. Y. Liu and B. N. Rome , “State Coverage and Reimbursement of Antiobesity Medications in Medicaid,” JAMA 331, no. 14 (2024): 1230–1232, 10.1001/jama.2024.3073. PMC10941017 38483403

[6] S. D. Sullivan , “Early Access to GLP‐1 Receptor Agonists for Weight Management in Medicare: The GLP‐1 Bridge Demonstration,” Annals of Internal Medicine 179, no. 9 (2026), 10.7326/ANNALS-26-01469. 42330504

[7] D. Witters , “In U.S., GLP‐1 Usage Reaches New High,” Gallup (2026), https://news.gallup.com/poll/712157/glp‐usage‐reaches‐new‐high.aspx.

[8] J. E. Raubenheimer , P. H. Myburgh , and A. S. Bhagavathula , “Sweetening the Deal: An Infodemiological Study of Worldwide Interest in Semaglutide Using Google Trends Extended for Health Application Programming Interface,” BMC Glob Public Health 2, no. 1 (2024): 63, 10.1186/s44263-024-00095-w. PMC11622965 39681910

[9] K. A. Gudzune , L. M. Kaplan , S. Kahan , et al., “Weight‐Reduction Preferences Among OBSERVE Study Participants With Obesity or Overweight: Opportunities for Shared Decision‐Making,” Endocrine Practice 30, no. 10 (2024): 917–926, 10.1016/j.eprac.2024.06.009. 38901731

[10] T. Graabæk , N. T. Nguyen , M. S. Sørensen , and C. Lundby , “‘This is My Decision’: A Qualitative Study of Individuals' Perspectives on Use of Semaglutide for Weight Loss,” Basic and Clinical Pharmacology and Toxicology 137, no. 1 (2025): e70069, 10.1111/bcpt.70069. PMC12168653 40521912

[11] C. K. Ibsen , M. Brostrøm Kousgaard , S. Olsen , et al., “Patients’ Experiences With GLP1‐RAs–A Systematic Review,” Scandinavian Journal of Primary Health Care 43, no. 2 (2025): 370–379, 10.1080/02813432.2025.2477141. PMC12090293 40071612

[12] S. Febrey , M. Nunns , J. Buckland , et al., “What are the Experiences, Views and Perceptions of Patients, Carers and Clinicians of Glucagon‐Like Peptide‐1 Receptor Agonists (GLP‐1 Ras)? A Scoping Review,” Health Expectations 28, no. 2 (2025): e70251, 10.1111/hex.70251. PMC11995417 40227008

[13] A. Montero , G. Sparks , M. Presiado , et al., “KFF Health Tracking Poll May 2024: The Public’s Use and Views of GLP‐1 Drugs,” Published May 10, 2024, https://www.kff.org/health‐costs/kff‐health‐tracking‐poll‐may‐2024‐the‐publics‐use‐and‐views‐of‐glp‐1‐drugs/.

[14] A. Tyson and E. Kikuchi , How Americans View Weight‐Loss Drugs and Their Potential Impact on Obesity in the U.S (Pew Research Center, 2024), https://www.pewresearch.org/science/2024/02/26/how‐americans‐view‐weight‐loss‐drugs‐and‐their‐potential‐impact‐on‐obesity‐in‐the‐u‐s/.

[15] I. Granados , D. E. Jake‐Schoffman , K. M. Governale , D. A. Fedele , and M. A. McVay , “Beyond the Individual: Family‐Level Impacts of Caregiver GLP‐1 Use on Food Environments and Child Health Behaviors,” Childhood Obesity (2026): 21532176261467610, 10.1177/21532176261467610. 42421417

[16] K. J. Pratt , S. Gonzalez‐Jordan , S. F. Noria , and J. A. Skelton , “Characteristics Associated With Risk and Protective Behaviors Among Children of Parents on Obesity Management Medications,” Academic Pediatrics 26, no. 6 (2026): 103345, 10.1016/j.acap.2026.103345. 42288152

[17] K. J. Pratt , S. Gonzlez‐Jordan , S. F. Noria , and J. A. Skelton , “Parental Communication With Children Around Obesity Management Medications, Weight Loss, and Health Behavior Change,” Obesity 34, no. 6 (2026): 1237–1242, 10.1002/oby.70206. 42036138

[18] S. D. Emmerich , C. D. Fryar , B. Stierman , and C. L. Ogden , “Obesity and Severe Obesity Prevalence in Adults: United States, August 2021–August 2023,” (NCHS Data Brief, 2024), no. 508, 10.15620/cdc/159281. PMC11744423 39808758

[19] R. Petersen , L. Pan , and H. M. Blanck , “Racial and Ethnic Disparities in Adult Obesity in the United States: Cdc’s Tracking to Inform State and Local Action,” Preventing Chronic Disease 16 (2019): E46, 10.5888/pcd16.180579. PMC6464044 30974071

[20] M. E. Vajravelu , P. Y. Chu , D. A. Frank , M. I. Ragavan , and R. K. Vajravelu , “Projected Impact of Anti‐Obesity Pharmacotherapy Use on Racial and Ethnic Disparities in Adolescent Obesity,” Pediatr Obes 19, no. 4 (2024): e13103, 10.1111/ijpo.13103. PMC12980788 38318987

[21] G. A. Woodard , B. Encarnacion , J. Peraza , T. Hernandez‐Boussard , and J. Morton , “Halo Effect for Bariatric Surgery: Collateral Weight Loss in Patients' Family Members,” Archives of Surgery 146, no. 10 (2011): 1185–1190, 10.1001/archsurg.2011.244. 22006878

[22] A. A. Gorin , E. M. Lenz , T. Cornelius , T. Huedo‐Medina , A. C. Wojtanowski , and G. D. Foster , “Randomized Controlled Trial Examining the Ripple Effect of a Nationally Available Weight Management Program on Untreated Spouses,” Obesity 26, no. 3 (2018): 499–504, 10.1002/oby.22098. PMC5838794 29388385

[23] K. J. Pratt , D. B. Sarwer , and J. A. Skelton , “Family Considerations in the Treatment of Adult Obesity With Antiobesity Medications,” Obesity 33, no. 9 (2025): 1619–1621, 10.1002/oby.24355. PMC12381599 40721288

[24] Temple Health 2025 , “Regional Community Health Needs Assessment for Southeastern Pennsylvania,” (2025), https://www.templehealth.org/sites/default/files/inline‐files/TempleUniversityHospital‐CHNA2025.pdf.

[25] A. Montero , A. Kearney , M. Mulugeta , et al., “KFF Health Tracking Poll: Prescription Drug Costs, Views on Trump Administration Actions, and GLP‐1 Use,” Published Nov 14, 2025, https://www.kff.org/public‐opinion/kff‐health‐tracking‐poll‐prescription‐drug‐costs‐views‐on‐trump‐administration‐actions‐and‐glp‐1‐use/.

[26] A. Montero , G. Sparks , A. Kirzinger , et al., “KFF Health Tracking Poll July 2023: The Public’s Views of New Prescription Weight Loss Drugs and Prescription Drug Costs,” Published Aug 4, 2023, https://www.kff.org/health‐costs/kff‐health‐tracking‐poll‐july‐2023‐the‐publics‐views‐of‐new‐prescription‐weight‐loss‐drugs‐and‐prescription‐drug‐costs/.

[27] E. S. Almughais , M. H. Alshehri , M. Alsatti , et al., “Awareness and Perception of Anti‐Obesity Medications Among Al‐Ahsaa, Riyadh, and Hail in Saudi Arabia Populations,” Cureus 15, no. 6 (2023), 10.7759/cureus.40425. PMC10348602 37456412

[28] W. Ghusn , A. De la Rosa , D. Sacoto , et al., “Weight Loss Outcomes Associated With Semaglutide Treatment for Patients With Overweight or Obesity,” JAMA Network Open 5, no. 9 (2022): e2231982, 10.1001/jamanetworkopen.2022.31982. PMC9486455 36121652

[29] M. H. Zeller , S. M. Robson , J. Reiter‐Purtill , et al., “Halo or Horn? A Qualitative Study of Mothers’ Experiences With Feeding Children During the First Year Following Bariatric Surgery,” Appetite 142 (2019): 104366, 10.1016/j.appet.2019.104366. PMC12481503 31301320

[30] S. E. Walters‐Bugbee , K. S. McClure , T. V. Kral , and D. B. Sarwer , “Maternal Child Feeding Practices and Eating Behaviors of Women With Extreme Obesity and Those Who Have Undergone Bariatric Surgery,” Surgery for Obesity and Related Diseases 8, no. 6 (2012): 784–791, 10.1016/j.soard.2012.07.016. 22940240

[31] A. S. Cleto , J. M. Schirlo , M. Beltrame , et al., “Semaglutide Effects on Safety and Cardiovascular Outcomes in Patients With Overweight or Obesity: A Systematic Review and Meta‐Analysis,” International Journal of Obesity 49, no. 1 (2025): 21–30, 10.1038/s41366-024-01646-9. 39396098

[32] V. Perkovic , K. R. Tuttle , P. Rossing , et al., “Effects of Semaglutide on Chronic Kidney Disease in Patients With Type 2 Diabetes,” New England Journal of Medicine 391, no. 2 (2024): 109–121, 10.1056/nejmoa2403347. 38785209

[33] A. M. Lincoff , K. Brown‐Frandsen , H. M. Colhoun , et al., “Semaglutide and Cardiovascular Outcomes in Obesity Without Diabetes,” New England Journal of Medicine 389, no. 24 (December 2023): 2221–2232, 10.1056/nejmoa2307563. 37952131

[34] P. Ueda , J. Söderling , V. Wintzell , et al., “GLP‐1 Receptor Agonist Use and Risk of Suicide Death,” JAMA Internal Medicine 184, no. 11 (2024): 1301–1312, 10.1001/jamainternmed.2024.4369. PMC11372654 39226030

[35] E. Kornelius , J.‐Y. Huang , S.‐C. Lo , C.‐N. Huang , and Y.‐S. Yang , “The Risk of Depression, Anxiety, and Suicidal Behavior in Patients With Obesity on Glucagon Like Peptide‐1 Receptor Agonist Therapy,” Scientific Reports 14, no. 1 (2024): 24433, 10.1038/s41598-024-75965-2. PMC11489776 39424950

[36] T. A. Wadden , G. K. Brown , C. Egebjerg , et al., “Psychiatric Safety of Semaglutide for Weight Management in People Without Known Major Psychopathology: Post Hoc Analysis of the STEP 1, 2, 3, and 5 Trials,” JAMA Internal Medicine 184, no. 11 (2024): 1290–1300, 10.1001/jamainternmed.2024.4346. PMC11372653 39226070

[37] US Food and Drug Administration , “FDA Requests Removal of Suicidal Behavior and Ideation Warning From Glucagon‐Like Peptide‐1 Receptor Agonist (GLP‐1 RA) Medications,” (January 2026), https://www.fda.gov/media/190557/download?attachment.

[38] B. Jankowska‐Polańska , I. Uchmanowicz , K. Dudek , and G. Mazur , Relationship Between Patients’ Knowledge and Medication Adherence Among Patients With Hypertension (Patient Pref Adherence, 2016), 2437–2447. 10.2147/PPA.S117269 PMC5153315 27994443

[39] C. H. Tae , S.‐A. Jung , H. S. Moon , et al., “Importance of Patients’ Knowledge of Their Prescribed Medication in Improving Treatment Adherence in Inflammatory Bowel Disease,” Journal of Clinical Gastroenterology 50, no. 2 (2016): 157–162, 10.1097/mcg.0000000000000431. 26501880

[40] D. Sherifali , M. Racey , D. Fitzpatrick‐Lewis , et al., “Missing the Target: A Scoping Review of the Use of Percent Weight Loss for Obesity Management,” Obesity Reviews 26, no. 11 (2025): e13960, 10.1111/obr.13960. PMC12531721 40461407

[41] S. Lee , N. Narula , M. Weglarz , J. Lee , and D. Kim , “Evaluation of Social Media Videos on Weight‐Loss Surgery and GLP‐1 Agonists: A Content Quality and Reliability Analysis,” Surgical Endoscopy 39, no. 9 (2025): 6032–6036, 10.1007/s00464-025-11938-4. PMC12408733 40634725

[42] S. Hristakeva , J. Liaukonytė , and L. Feler , “The No‐Hunger Games: How GLP‐1 Medication Adoption is Changing Consumer Food Demand,” Journal of Marketing Research 63, no. 3 (2026): 427–444, 10.1177/00222437251412834.

[43] K. J. Pratt and J. A. Skelton , “Family Functioning and Childhood Obesity Treatment: A Family Systems Theory‐Informed Approach,” Academic Pediatrics 18, no. 6 (2018): 620–627, 10.1016/j.acap.2018.04.001. PMC8111666 29654905

