# Supplementary material for: Patient Knowledge of GLP‐1 Receptor Agonists for Obesity and Exploration of Potential Household‐Level Effects in an Urban Health System
Source: Obes Sci Pract. 2026 Oct 7;12(5):e70199. doi: 10.1002/osp4.70199 (PMC13646696; doi:10.1002/osp4.70199)
Supplement: Supplementary file 1 [file OSP4-12-e70199-s001.docx]

**Supplemental Materials**

Supplementary Figure S1. Study Flow Diagram

Completed screener survey

n = 93

Responses considered invalid & removed

- Duplicate survey, n = 7
- Completed too quickly, n = 4

Sample analyzed

n = 52

Completed full survey

n = 63

Consented to participate

n = 71
